# Study pressure and self harm in Chinese primary school students: the effect of depression and parent-child relationships

**DOI:** 10.3389/fpsyt.2025.1580527

**Published:** 2025-04-30

**Authors:** Jing-Lan He, Xiao-Ming Xu, Wo Wang, Jian-Mei Chen, Qi Zhang, Yao Gan, Jun Cao, Da-Qin Ding, Jin-Hui Hu, Xiaorong Chen, Li Kuang

**Affiliations:** ^1^ Department of Psychiatry, The First Affiliated Hospital of Chongqing Medical University, Chongqing, China; ^2^ Psychiatric Center, The First Affiliated Hospital of Chongqing Medical University, Chongqing, China; ^3^ Mental Health Center, University-Town Hospital of Chongqing Medical University, Chongqing, China

**Keywords:** self harm, study pressure, depression, parent-child relationships, primary school students

## Abstract

**Background:**

Self-harm has become a significant and increasing global problem in children and adolescents. In this study, we tested a moderated mediation model to examine the impact of study pressure and depression, and one protective factor, good parent-child relationship, on self harm.

**Methods:**

A self-report Questionnaire survey was conducted among 33,285 primary school students from 3rd-6th grade (mean age = 10.36 years, SD = 1.24, 51.7% girls) in Shapingba District Chongqing, China. The survey assessed study pressure, depression, parent-child relationship, and self harm behaviors.

**Results:**

The reporting rates of self-harm and depression in grades 3-6 of primary school were 12.7% and 16.3%, respectively. Both moderate and high study pressure were significantly associated with an increased risk of self-harm, with depression acting as a mediator (indirect effects: moderate study pressure = 0.045, high study pressure = 0.125, p < 0.001). Furthermore, a good parent-child relationship weakened the association between study pressure and self-harm, thus self harm on moderate study pressure (β=-0.057) and on high study pressure (β=-0.032) are lower than those without the moderator of a good parent-child relationship(β=0.116; β=0.168), as well as between study pressure and depression.

**Conclusions:**

This study is the first to conduct a self-harm survey in the largest population-based sample of Chinese primary school students. The results highlight the importance of monitoring study pressure, fostering a positive parent-child relationship, and managing depressive symptoms to support students’ mental health. These findings enhance our understanding of the development of self-harm behaviors and can inform the design of targeted intervention programs aimed at reducing self-harm among primary school students.

## 1 Introduction

Self-harm (including intentional self-poisoning or self-injury, regardless of suicidal intent or underlying motives) refers to a range of behaviors such as suicide attempts and non-suicidal self-injury (1, 2). It is the third leading cause of disability-adjusted life-years (DALYs) among adolescents aged 10-24 years (3). Self-harm is a growing global concern in children and adolescents, with significant associations with future suicide risk. Beyond suicidal behavior, self-harm can also serve as an early indicator of psychiatric disorders, including psychosis and depression in adulthood (4). According to international surveys, approximately 14% of children and adolescents report engaging in self-harm (1). A meta-analysis of global community-based studies found that the lifetime prevalence of self-harm in adolescents was 16.9% (95% CI 15.1-18.9), with rates increasing from 1990 to 2015 (5). Despite substantial research on self-harm in adolescents and young adults, studies focusing on younger children, particularly primary school students, remain limited.

Conducting a comprehensive, large-scale survey on the mental health of primary school students in China presents considerable challenges, hence there were a limited number of studies on self-harm behaviors among primary school students. Other studies tended to have smaller sample sizes, ranging from 352 to 4777 (6). Notably, a recent survey in China found that the prevalence of non-suicidal self-injury in primary school students (29.3%) was higher than in middle school students (25.3%) (6). This highlights the need for greater attention to self-harm in children under 12 years old. Additionally, children hospitalized in psychiatric wards or pediatric intensive care units (PICUs) further underscore the severity and universality of self-harm. In fact, 37% of children admitted for neuropsychiatric events were diagnosed with self-harm (7). The strong association between self-harm and suicidal tendencies further emphasizes the need for research into the risk and protective factors that could inform interventions to mitigate both self-harm and suicide rates in children.

The factors that contribute to self harm in young people, especially mechanisms underlying contagion, remained major challenges. Important contributors to self-harm include genetic vulnerability and psychiatric, psychological, familial, social, and cultural factors (1). For primary school students, psychiatric, familial and school factors played more prominent roles among all these factors.

Study pressure seems a common problem for students all over the world and positively associated with at least one mental health outcome within the school year (8). Studying problems was the third prevalent life problems in younger children, only after the relationship with family members and relationships with friends, especially among those who repeated self harm (9). A higher proportion of children and young people up to the age of 24 years with a self-harm record were absent from school or excluded in any school year compared with pupils without a record (10). Thus on the contrary worsening the study pressure in children. In a global study, both the lifetime and 12-month aggregate prevalence of non-suicidal self-injury were higher in the school-attending group as compared to the partial and non-school attending group (22.8% vs. 19.0%), and (21.5% vs. 8.0%) respectively. So as the aggregate lifetime and 12-month prevalence of deliberate self-harm (15.3% vs. 10.4%) (11). Most studies investigated the association between performance or timing within the school year and mental health outcomes, while little study directly assessed the association between study pressure and self harm, especially in primary school students (8).

Depression in primary school students has become a public health issue of concern. Risks including self harm, suicide and self-neglect increased from low mood to depressive disorder (12). Prevalence rates of current depression are reported to be 3.2% among children aged 3-17 years in US (13). While the pooled prevalence of depressive symptoms in Chinese primary school students was 17.2% (95% CI: 14.3%-20.5%), with western China reporting the highest prevalence (14). Anxiety, depression, and other mood disorders characterized the high-risk profile of self harm in both female and male children hospitalized with a neuropsychiatric event (7). Persistent symptoms of depression is the antecedent (waves 1-3) predictors of self-harm in primary school-aged children (15). Children with depression typically have functional impairments in their performance at school as well as in their interactions with their families. Depression in children is strongly associated with recurrent depression in adulthood; other mental disorders; and increased risk for suicide (16). Stressful life events, like study pressure, could induce a series of psychological and physiological changes including structural and neurochemical changes of brain regions, endocrines and immune systems dysfunction which were closely related to depression (17). Environmental stress and early childhood adversity, can alter biological systems via epigenetic mechanisms and have long-lasting consequences, increasing risk for negative mental health outcomes, including depression (18). Therefore, we hypothesized depression as a mediator between study pressure and self harm.

Parent-child relationship within the family is important for the development of children’s well-being. Parental factors were strongly associated with childhood self-harm, with over three-quarters of children with self-harm having a parent with a history of mental disorder and/or criminal offending (19). Bad parent-child relationship may increase the risk of children self harm behaviors. The likelihood of deliberate self-harm was 49 per cent and 61 per cent significantly more likely among adolescent boys and girls who experienced parental physical abuse respectively (20). Higher parent-child relationship quality is associated with lower levels of adolescent depression (21, 22). Depression was positively associated with study problems only or with parent-child relationship problems in participants (23). On the contrary, parental support was related to lower self-harm prevalence among adolescents and Eastern parents have more influence on adolescents’ behaviors (24). Parenting factors that have been found to mediate program effects on youth outcomes of depression and anxiety in young people include parental warmth, authoritative parenting, effective and consistent discipline, parental monitoring, and good family communication and problem solving (25). Positive family communication and support and parental involvement may work as protective against adolescent self-harm (26). Theoretical frameworks such as the Family Stress Model posit that supportive family relationships may buffer the negative effects of external stressors (e.g., academic pressure) on mental health outcomes (27). In the context of Chinese primary school students, where parental involvement in education is intensive (28), the quality of parent-child relationships is likely to act as a boundary condition that alters the strength of the pathway from study pressure to depression and subsequent self-harm. This aligns with empirical evidence showing that parental warmth moderates the association between school stress and emotional dysregulation in children (29). Therefore, we hypothesize that a good parent-child relationship serves as a moderator, attenuating both the direct effect of study pressure on self-harm and its indirect effect via depression.

From the aforementioned, we knew that previous studies established preliminary associations between study pressure, depression, family relationship and self harm. Despite extensive studies on adolescents, little is known about the risk or protective factors of self harm and the relationship of the factors in children. The present study constructed a moderated mediation model in the largest population-based sample of Chinese primary school students by including study pressure and depression as two risk factors, and good parent-child relationship as one protective factor, to investigate the underlying mechanisms of self harm in children. More specifically, we proposed the following hypotheses (1): Study pressure and depressive symptoms would be positively associated with self-harm among primary students (2); depression would mediate the relationship between study pressure and self harm among primary students (3); parent-child relationship would moderate the indirect and direct relationships between study pressure and self harm among primary students through depression, such that good parent-child relationship would mitigate the indirect and direct impacts of study pressure on children self harm through depression.

## 2 Method

This study employed a cross-sectional survey design, screening the mental health data of primary school students in District S of Chongqing during the period from September to December 2020. Data collection was suspended during school exams and holidays to ensure the accuracy and reliability of the results. The survey was supported by the schools and teachers in the district. Both the participants and their guardians provided informed consent.

Head teachers, school psychologists, and computer teachers received specialized training to ensure the smooth progress of the survey. During the data collection process, these trained personnel and the research team assisted with any queries related to the survey questionnaires and kept order at each site. In addition, a technical support team, including engineers, was available to address any challenges encountered during the management of the online survey.

### 2.1 Participants

Participants were recruited from 3rd to 6th graders primary schools in Chongqing, southwestern China, through cluster sampling. A total of 33,895 students ranging in age from 7 to 14completed questionnaires in their classrooms. Students in grades 1-2 may have biases in understanding the self-assessment questionnaire items, and the applicable age for the questionnaire does not match, so they were not included in the study.

### 2.2 Questionnaire survey

The basic information such as school, grade, sex, age, and student ID, were collected using a structured e-questionnaire.

Considering the patience and understanding level of primary school students, the assessment scales applicable to primary school students were limited. Therefore, we used simple multiple-choice questions to assess the self harm behaviors, study pressure and parent-child relationship of primary school students.

#### 2.2.1 Study pressure

In this study, study pressure was designed as the exposure variable, investigating its relationship with self-harm. Study pressure was assessed using an optional question, “How would you feel your study stress?” with the response options response “0. None” or “1. Moderate” or “2. High”. Study pressure was operationalized as students’ perceived burden from academic demands, including workload, examination expectations, and family/school performance pressures. This question was designed based on common stressors in Chinese primary education contexts. To ensure clarity, the term “study pressure” was explained by teachers during survey administration. Pretesting confirmed the question’s age-appropriateness and comprehensibility.

#### 2.2.2 Depression

Depression was the mediation variable, investigating its mediation effect between study pressure and self-harm. It was assessed by using the Children’s Depression Inventory (CDI) (30). The CDI score was calculated by assigning ratings of 0, 1, and 2 to the response categories of “sometimes”, “often”, and “always”, respectively. Children were instructed to select the best response that described how they felt over the last two weeks. A CDI score of 19 was used as the cutoff for depression (31). The Cronbach’s alpha for CDI in this study was 0.884, indicating good internal consistency.

#### 2.2.3 Parent-child relationship

Parent-child relationship was the moderator and assessed using a optional question, “How is your relationship with your parents? “ with the response options response “1. Good 2. Average 3. Poor”. In this study, we would like to explore the protective factors against self-harm among primary school students. Therefore, we classify the parent-child relationship into a dichotomous variable of “good” and “not good”, where “not good” includes the “2.average” and “3.poor” categories in the questionnaire.

#### 2.2.4 Self harm

Self harm behaviors were the outcome and assessed using an optional question, “Have you ever hurt yourself when you were unhappy?” with the response options response “Yes” or “No”.

### 2.3 Covariates

Gender (male/female), age were used as covariates in the analyses. These variables have all been shown to be associated with risk of self harm.

### 2.4 Procedure

This research obtained ethical approval from both the Chongqing medical University and Chongqing Shapingba Education Commission as a mental health screening initiative for primary school students. Informed consent was obtained from teachers, parents, and participating students prior to information collection. Participating students spent about 30 min to complete a series of self-report questionnaires online in their computer class. The results were securely exported to a backend system and encrypted. To promote honest responses, participants were assured that their answers would remain strictly confidential. They were also informed that their participation was voluntary and that they could withdraw from the study at any time.

### 2.5 Statistical analyses

SPSS 25.0 version was used to generate descriptive statistics and correlations. Mplus 8.1 was used to conduct structural equation modeling using maximum likelihood estimation and bias-corrected percentile bootstrapping with 5,000 replications. The analyses tested whether the association between study pressure and self harm was mediated by symptoms of depression. They also tested whether parent-child relationship moderated (weakened) this indirect association. Specifically, parent-child relationship was tested as a moderator of the link between study pressure and depression; moderation in any link constitutes moderation of the whole mediation model. We controlled for gender and age as predictors in regression equations as part of structural equation modeling.

The study gained ethics approval via the Ethics Committee of the First Affiliated Hospital of Chongqing Medical University for Health and Human Sciences (2020–879).

## 3 Results

### 3.1 Descriptive analysis

The final sample included 33,285 students (51.72% girls, Mage = 10.36 years) after excluding 205 incomplete responses, for a response rate of 98.8%. The sample comprised 5,229 third graders, 9,668 fourth graders, 9,526 fifth graders and 8,862 sixth graders. The students resided in both rural (17%) and urban (83%) areas. **Table 1** presents the demographic statistics of the participants.

**Table 1:** Demographic statistics of the participants.

Variables	N/Mean	%/Standard Deviation
Age	10.36	1.24
Sex		
Male	16071	48.3
Female	17214	51.7
Self-harm		
No	29062	87.3
Yes	4223	12.7
Depression		
No	27854	83.7
Yes	5431	16.3
Parent-child relationship		
Not good	32959	99.1
Good	326	0.9
Study pressure		
High	3174	9.5
Moderate	16876	50.7
Low	13235	39.8

We used the SPSS statistical software to calculate the Phi coefficient through the cross-table (Crosstabs) method and evaluate the statistical significance combined with the chi-square test (Chi-square test). The results of this study can be used to reveal potential associations between dichotomous variables for further analysis. **Table 2** presents the frequency, proportion, and correlations for the measured variables. As expected, study pressure, depression and self harm were significantly and positively correlated with each other (p<0.01). Parent-child relationship was significantly and negatively correlated with the other variables (p<0.01).

**Table 2:** Descriptive statistics and correlations of the study variables.

	1	2	3	4	5
1.self harm	-				
2.depression	0.38^**^	-			
3.moderate study pressure	0.03^**^	0.01^*^	-		
4.high study pressure	0.24^**^	0.34^**^	-0.33^**^	-	
5.parent-child relationship	-0.22^**^	-0.33^**^	-0.06^**^	-0.23^**^	-
frequency	4223	5431	16876	3174	27698
Proportion(%)	12.7	16.3	50.7	9.5	83.2

^*^ *p*<.01, ^**^ *p*<.01.

### 3.2 Testing for mediation effect of depression

**Figure 1:**
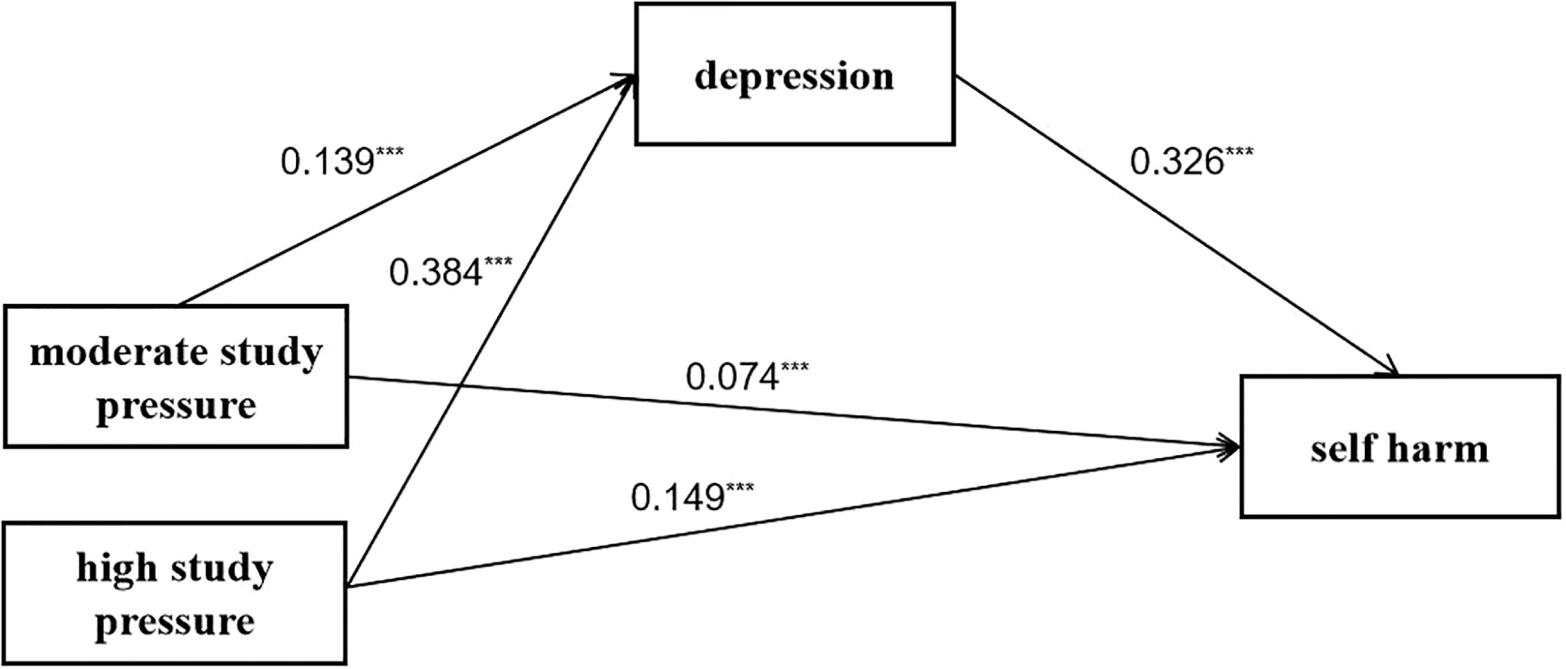
The mediating effect of depression in the association between study pressure and self harm. ***p <.001.

Next, we tested the mediation effect of depression between study pressure and self harm in **Figure 1** and **Table 3**. The mediation effect is considered significant if the confidence interval (CI) does not include 0. After controlling for sex and age, moderate study pressure was positively associated with depression (β=0.139, SE=0.005, t=29.763, p<0.001) and self harm (β=0.074, SE=0.005, t=15.657, p<0.001), while high study pressure was also positively associated with depression (β=0.384, SE=0.005, t=52.308, p<0.001) and self harm (β=0.149, SE=0.006, t=19.508, p<0.001), and depression was positively associated with self harm (β=0.326, SE=0.005, t=43.306, p<0.001).

**Table 3:** The mediating role of depression.

Dependent variable	Predictor	β	SE	T-value	P-value
Self harm	depression	0.326^***^	0.005	43.306	^***^<0.001
	moderate study pressure	0.074^***^	0.005	15.657	^***^<0.001
	high study pressure	0.149^***^	0.006	19.508	^***^<0.001
Depression	moderate study pressure	0.139^***^	0.005	29.763	^***^<0.001
	high study pressure	0.384^***^	0.005	52.308	^***^<0.001

Adjustment for gender and age.

^***^p<.001.

Depression partially mediated the relationship between study pressure and self harm, according to biascorrected bootstrapping results on moderate study pressure (indirect effect=0.045, t=24.182, CI=[0.04, 0.05]) and on high study pressure (indirect effect=0.125, t=33.157, CI=[0.12, 0.13]). As shown in **Table 4**, the mediation effect on moderate study pressure accounted for 38% of the total effect (β=0.119, t=24.667, p<0.001, CI=[0.11, 0.13]) while high study pressure accounted for 46% of the total effect (β=0.274, t=36.003, p<0.001, CI=[0.26, 0.29]).

**Table 4:** The mediation effects in direct and indirect effects of the model.

Mediating variable	Effect	β	95% CI	SE	Effect ratio
depression					
From X2 to Y	total effect	0.119^***^	(0.11, 0.13)	0.006	
	direct effect	0.074^***^	(0.06, 0.08)	0.002	
	indirect effect	0.045^***^	(0.04, 0.05)	0.005	38%
From X3 to Y	total effect	0.274^***^	(0.26, 0.29)	0.005	
	direct effect	0.149^***^	(0.13, 0.17)	0.006	
	indirect effect	0.125^***^	(0.12, 0.13)	0.003	46%

Adjustment for gender and age.

X2: moderate study pressure. X3: high study pressure. Y: self harm.

^***^p<.001.

### 3.3 Testing for moderated mediation effect of parent-child relationship

In the analysis of the regulatory effects in this study, we used multiple regression analysis and introduced an interaction term to examine the regulatory effect of different parent-child relationships (Good, Average, Poor) on the study pressure-self harm relationship. First, we converted the parent-child relationship variables into dumb variables (Average, Poor, based on Good), and mean-centered the independent variable study pressure to reduce the multicollinearity problem. Subsequently, we used hierarchical regression analysis to construct the main effect model and the interaction effect model, test the effect of study pressure, Average, Poor and their interaction term (study pressure×Average, study pressure×Poor) on self harm, and compared the Δ R² change to determine the significance of the regulatory effect.

The two models were shown in **Table 5**. The regulatory effect of Model 1 was significant (p <0.001), indicating that a good parent-child relationship does play a regulatory role in the relationship between study pressure and self harm. The Δ R² of Model 2 is very small (0.001), indicating that the actual influence of the interaction term is low, that is, although the regulatory effect exists, its contribution to the overall model is weak. R² did not improve much (18.6% 18.7%), indicating that the main effect still came from the main effect of study pressure and parent-child relationship, rather than their interaction.

**Table 5:** The two models of the regulatory effect of different parent-child relationships.

Model	R²	Adjusted R²	ΔR²	F Change	Sig. F Change
Model 1	0.186	0.186	0.186	2534.77	<0.001
Model 2	0.187	0.187	0.001	15.74	<0.001

Model 1:Main effects only-Study pressure(X) and High parent-child relationship (moderator variable).

Model 2:Add an interaction term - study pressure(X)*poor parent-child relationship and study pressure(X)*average parent-child relationship.

**Figure 2:**
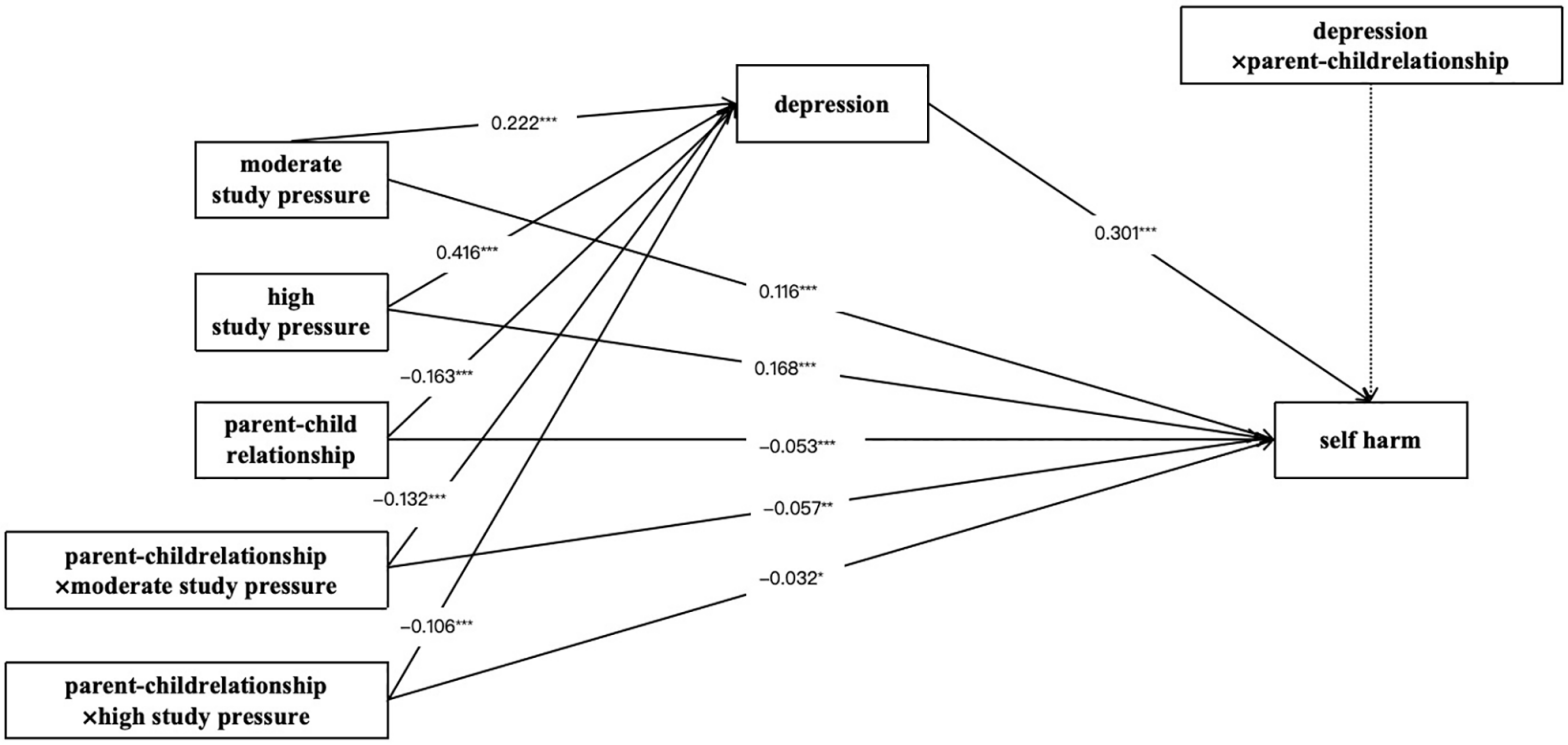
The moderating effect of parent-child relations on the direct and indirect associations between study pressure and self harm.^*^ *p*<.05, ^**^ *p* <.01, ^***^ *p* <.001.

Then, we tested the moderated mediation effect of parent-child relationship in **Figure 2**. The moderation effect is considered significant if the confidence interval does not include 0. The results showed that moderate study pressure (β=0.116, SE=0.019, p<0.001), high study pressure (β=0.168, SE=0.015, p<0.001), depression (β=0.301, SE=0.008, p<0.001) and parent-child relationship (β=-0.053, SE=0.012, p<0.001) were significantly associated with self harm. Moderate study pressure (β=0.222, SE=0.021, p<0.001), high study pressure(β=0.416, SE=0.014, p<0.001) and parent-child relationship (β=-0.163, SE=0.013, p<0.001) were significantly associated with depression. More importantly, parent-child relationship significantly moderated the impacts of moderate study pressure on depression (β=-0.132, SE=0.021, p<0.001) and self harm (β=-0.057, SE=0.020, p=0.004). In a similar vein, the effects of high study pressure on depression (β=-0.106, SE=0.013, p<0.001) and self harm (β=-0.032, SE=0.013, p=0.016) were considerably mitigated by parent-child relationship.

Under the condition of good parent-child relationship, the probability of depression on moderate study pressure (β=-0.132) and depression on high study pressure (β=-0.106) are lower compared with those without being moderated (β=0.222; β=0.416), which indicates that a good parent-child relationship can reduce the depression of primary school students caused by moderate or high study pressure. Similarly, the likelihood of self harm on moderate study pressure (β=-0.057) and self harm on high study pressure (β=-0.032) are also lower than those without the moderator (β=0.116; β=0.168). In other words, good parent-child relationship can also reduce the self harm of primary school students caused by moderate or high study pressure.

Finally, we further estimated the direct and indirect effects of the model. As indicated in **Table 6**, the indirect effect between moderate study pressure and self harm via depression was lower on good parent-child relationship (indirect effect=0.018, SE=0.001, t=16.910, p<0.001), than those without the moderator (indirect effect=0.044, SE=0.004, t=10.081, p<0.001). The indirect impact of depression on high study pressure was also lower on good parent-child relations (indirect effect=0.094, SE=0.004, t=22.950, p<0.001), than when there was no moderator (indirect effect=0.142, SE=0.006, t=23.213, p<0.001). Similarly, the direct effect of study pressure on self harm varied with the level of parent-child relationship. The direct effect of moderate study pressure (direct effect=0.039, SE=0.003, t=12.115, p<0.001) and high study pressure (direct effect=0.143, SE=0.010, t=13.796, p<0.001) were lower on good parent-child relationship compared with those without being moderated (direct effect=0.077, SE=0.013, t=5.972, p<0.001; direct effect=0.190, SE=0.017, t=11.117, p<0.001).

**Table 6:** The moderation effects in direct and indirect effects of the model.

Variable	Estimate	SE	T-value	P-value
IND X2_NW	0.044^***^	0.004	10.081	<0.001
IND X2_WW	0.018^***^	0.001	16.910	<0.001
IND X3_NW	0.142^***^	0.006	23.213	<0.001
IND X3_WW	0.094^***^	0.004	22.950	<0.001
DIR X2_NW	0.077^***^	0.013	5.972	<0.001
DIR X2_WW	0.039^***^	0.003	12.115	<0.001
DIR X3_NW	0.190^***^	0.017	11.117	<0.001
DIR X3_WW	0.143^***^	0.010	13.796	<0.001
TOT X2_NW	0.121^***^	0.014	8.946	<0.001
TOT X2_WW	0.056^***^	0.003	17.025	<0.001
TOT X3_NW	0.332^***^	0.017	19.008	<0.001
TOT X3_WW	0.238^***^	0.011	21.871	<0.001

Adjustment for gender and age.

IND: indirect effect. DIR: direct effect. TOT: total effect. X2: moderate study pressure. X3: high study pressure. NW: without the moderator. WW: with the moderator.

^***^ *p*<.001.

## 4 Discussion

Primary school students exhibit a high rate of self harm behavior, which is closely related to school, family, and personal factors. This study firstly conducted a self harm survey in the largest population-based sample of Chinese primary school students. Study pressure and depression were found to be positively associated with self harm, while parent-child relationships were negatively associated. Depression mediated the connection between study pressure and self harm. Additionally, a good parent-child relationship weakened the association between study pressure and self harm, as well as between study pressure and depression.

First, this study found that the incidence of self-harm among primary school students was 12.7%. This result is consistent with the global average incidence rate of self-harm among children and adolescents reported in community and school surveys (14%) (32), but significantly lower than the 29.3% incidence rate reported in previous studies of Chinese elementary school students (6). This discrepancy may be attributed to the definition of self-harm, types of self-harm, different time frames of incidence and the characteristics of the study populations. Typically, the incidence rate of suicide attempts (1.3%) is much lower than that of non-suicidal self-injury (21.9%) (2), highlighting the importance of distinguishing between different types of self-injurious behavior. The aggregate lifetime and 12-month prevalence of non-suicidal self-injury was 22.1% and 19.5% while deliberate self-harm was 13.7% and 14.2% respectively (11). Furthermore, the incidence rates of self-harm reported in community and school surveys are generally lower than those from studies conducted in clinical settings such as pediatric or emergency departments (37%) (7), which may be due to selection bias in the study samples. In clinical environments, the samples often include individuals with more severe manifestations of the behavior, leading to higher reported rates of self-harm. The varying research contexts and definition criteria may significantly impact the reported incidence of self-harm, and future studies should aim to clarify and standardize these definitions and criteria.

We directly assessed the association between study pressure and self harm, and found study pressure was significantly and positively correlated with self harm. According to the Erikson’s psychosocial theory, School Age period is the critical period of “industry vs. inferiority” for primary school students to develop competence in which study pressure has a significant impact on them. If primary school students repeatedly experience setbacks in their studies, they may develop a sense of inferiority and lose interest in learning. This can affect their future sense of self-efficacy and self-esteem and have a profound impact on their future study. Reasonable study pressure in school, with praise for their accomplishments would reduce maldevelopment of inertia or passivity which might lead to increased psychological pressure and self harm (33). Most previous studies focus on middle school and college students. Research on adolescent populations has found that study pressure is associated with mental states such as anxiety and depression. Regular observation of school time reveals that there is a decrease in self harm during winter and summer vacations, but there is little directly assessed the association between study pressure and self harm (8). In our study, there were still 60.2% primary school students who experienced study pressure and 9.5% experienced high study pressure. The issue of study pressure among primary school students may be underestimated. Higher study pressure (β=0.149) the primary students felt was more positively associated with self harm compared to moderate study pressure(β=0.074). The greater the study pressure, the more self harm behaviors primary school students exhibited. Attention should be paid to the level of study pressure and academic performance among primary school students, and those with high levels of study pressure should be guarded against potential adverse mental health issues such as self-harm.

Our findings supported the hypothesized model in which depression mediates the association between study pressure and self harm in Chinese primary school students. In those who beard high study pressure, the indirect effect accounted for 46% of the total effect. Current researches mainly focused on exploring the direct relationship between study pressure and depression, or between depression and self-harm (6, 15), consistently finding that depressive symptoms are closely related to study pressure and self harm (34). However, these studies have paid less attention to the potential mediating role of depression between the two variables. A small number of studies have suggested that the mediating role of depressive symptoms varies depending on the research context and risk factors. For example, in studies on non-suicidal self-injury related to family intimacy and family adaptability, the mediating effect values of depressive symptoms are -0.0029 and -0.0071 (35), respectively, which are much lower than the mediating effects found in this study under moderate study pressure (0.045) and high study pressure (0.125). In contrast, research on cyberbullying and NSSI (Non-Suicidal Self-Injury) shows that the mediating indirect effect of depression is 0.14, accounting for 60.8% of the total indirect effect (36). In studies on the impact of family environment on NSSI, the indirect effect of depression reaches 0.0318, accounting for 64.64% of the total effect (37). These research findings were consistent with this study, further supporting the significant mediating role of depressive symptoms in self harm among children and adolescents. Study pressure may cause student school burnout which also looks as if depressed, like a sense of ineffectiveness and lack of accomplishment (38), which in turn affect primary school students’ performance and self-efficacy at school, reduce their life satisfaction (39), and ultimately increase the risk of self-harm behavior (40). Under conditions of high study pressure, the proportion of the mediating effect of depressive symptoms significantly increases to 46%, much higher than the 38% under general study pressure levels. This indicates that the greater the study pressure the primary school students have, the more severe the depressive emotions they may have, leading to a significant increase risk of self harm. It is evident that the depressive emotions of primary school students should be taken very seriously, especially for those under greater study pressure. Their emotional states should be closely monitored, and emotional regulation strategies should be actively taught to reduce the incidence of self harm.

In our study, good parent-child relationship had a significant protective effect among primary school students, reducing self-harm behaviors triggered by moderate study stress and high study stress. Under conditions of moderate study stress, the moderating role was more pronounced. Parent-child relationship plays a complex moderating role between study pressure and depression and self harm. Its effects can be both protective and risking. Previous studies had largely focused on risk factors for self harm, such as indicating that poor family relationships can exacerbate self harm behaviors (41–43). This study, however, focuses on protective factors for self harm behaviors among primary school students, and the results showed that a good relationship with parents could reduce the probability of depression caused by study pressure in primary school students, which in turn leaded to a lower likelihood of self harm. This indicated that a good relationship with parents plays a key moderating role in alleviating depressive symptoms and self harm behaviors. Parents can help children better tolerate and manage unpleasant feelings (44), and primary school students with a good relationship with their parents also feel less academic expectations and pressure from their parents, resulting in fewer depressive emotions, thereby reducing the impact of study pressure on self harm. Moreover, by categorizing study pressure into moderate and high levels, we can better comprehend the scope of pressure that primary school students encounter, thereby offering insights for the formulation of targeted intervention strategies at different stages. This study found that during the stage of moderate study pressure, the moderating effect of a good parent-child relationship on reducing self harm behaviors in primary school students was stronger; however, when study pressure increased and lead to significant emotional dysregulation and self harm behaviors, the moderating effect of the parent-child relationship was somewhat diminished. This indicated that in the initial phases of study pressure, parents should be more proactive in assisting primary school students to manage their emotions and alleviate pressure to prevent the onset of self harm, rather than waiting until study pressure accumulates to a more severe degree before intervening.

However, this research still has limitations that can be improved in future studies. Firstly, although Chongqing is a centrally-administered municipality, it is located in the southwestern region of China and is slightly less economically developed than the eastern regions of the country. This study comprehensively surveyed primary school students in a single administrative district, covering both urban and rural areas, which can represent the family situations across different economic levels. Additionally, this administrative district is an important educational area in Chongqing, encompassing key schools, ordinary schools, and rural schools, thereby representing the conditions of schools with varying levels of educational quality. The study covered a large sample size with 33,285 primary school students in Chongqing province, but the potential differences, such as geographical differences, racial differences and so on, still need to be adequately accounted. Multi center researches can be conducted in the future to further reduce potential differences. Due to the unique characteristics of primary school students’ comprehension abilities and the age restrictions of the questionnaire, the conclusions of this study cannot be fully generalized to adolescents and adults. They are limited to reference within the study population. Secondly, this study used rigorous statistical analysis, cross-sectional mediation provides an indication for, but not proof of the presence of an underlying mechanism. The mediation role of depression and the moderated mediation effect of parent-child relationship revealed the correlation between study stress and self harm, but its mechanism was still unclear. We still need to conduct longitudinal studies to confirm the underlying mechanism. Finally, the assessment scales applicable to primary school students were limited. We used simple multiple-choice questions to assess the self harm behaviors, study pressure and parent-child relationship. The use of self-report instruments could affect the quality and validity of the participants’ answers. Replicating similar studies with experimental and causal-comparative methods, using scales with verified reliability and validity, or face-to-face interview can provide more reliable results.

## 5 Conclusion

This is the first time to conduct a self harm survey in the largest population-based sample of Chinese primary school students. This study revealed that study pressure, depression, and parent-child relationships were significantly associated with self-harm behaviors among primary school students. Depression was found to mediate the relationship between study pressure and self harm, especially in those who beard high study pressure. Notably, a good parent-child relationship significant reduced self-harm behaviors triggered by moderate study stress and high study stress, especially under conditions of moderate study stress. These findings contribute to our understanding of the development of self-harm behaviors and can inform the design of targeted intervention programs aimed at reducing self-harm among primary school students. We need to pay attention to the study pressure experienced by primary school students and reduce excessive study pressure and the resulting depression. For primary school students who have already developed depression, it is important to actively teach them emotional regulation strategies to reduce self harm. Parents should also help reduce depression and self harm in their children by establishing good parent-child relationships when the children are under study pressure in the initial phases.

## Data Availability

The datasets presented in this article are not readily available because the data is sourced from primary school students and is not suitable for sharing. Requests to access the datasets should be directed to Jing-Lan He, 381380757@qq.com.
