# Supplementary material for: Study pressure and self harm in Chinese primary school students: the effect of depression and parent-child relationships
Source: Front Psychiatry. 2025 Apr 30;16:1580527. doi: 10.3389/fpsyt.2025.1580527 (PMC12076088; doi:10.3389/fpsyt.2025.1580527)
Supplement: Supplementary file 1 [file DataSheet1.docx]

Supplementary Material

# Supplementary Figures


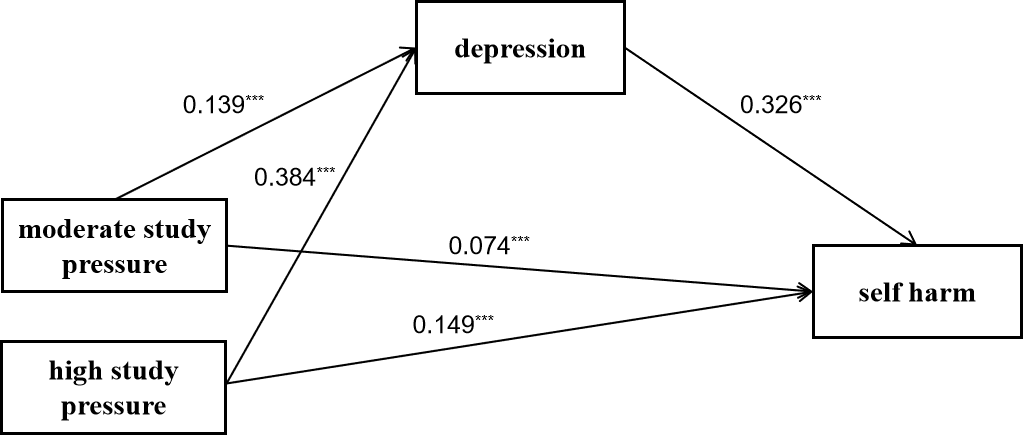


Figure 1 The mediating effect of depression in the association between study pressure and self harm. ****p* < .001.


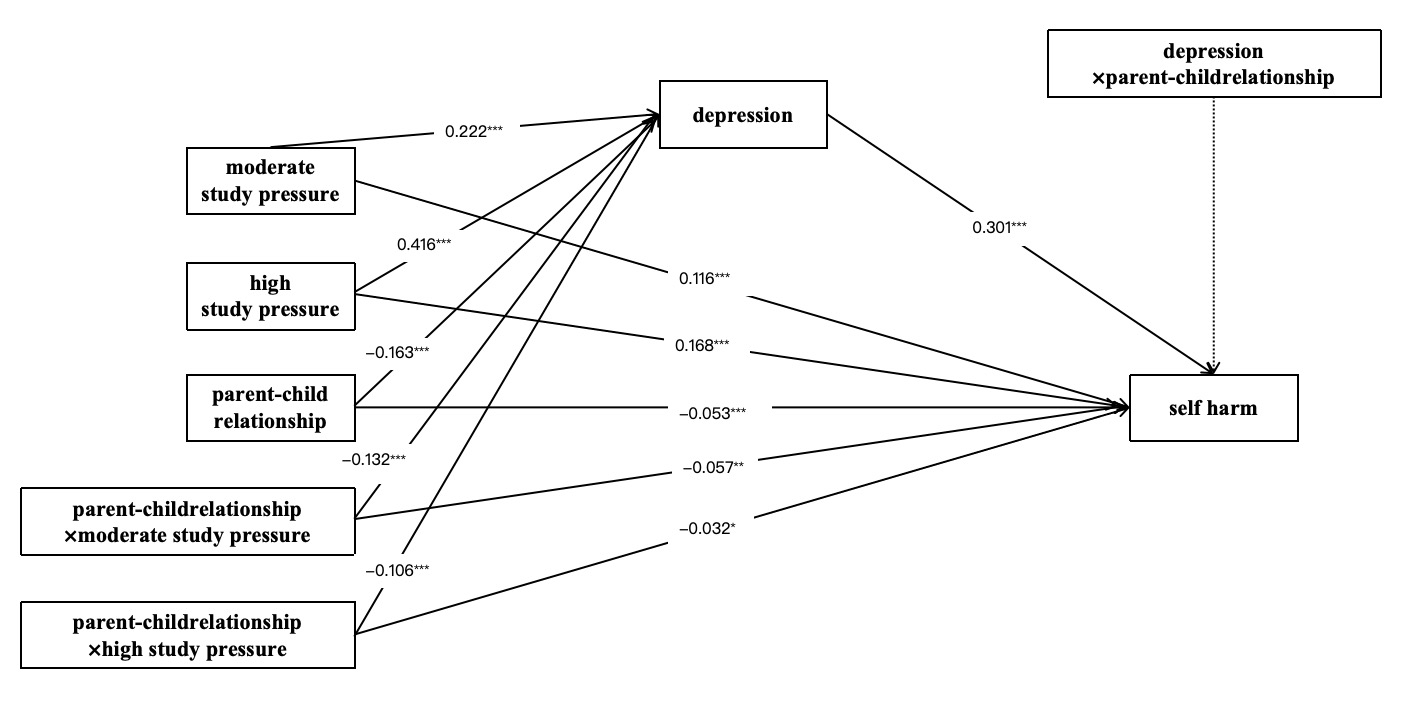


Figure 2 The moderating effect of parent-child relations on the direct and indirect associations between study pressure and self harm.**p*<.05, ***p* < .01, ****p* < .001.
